# Beneficial and Detrimental Remodeling of Glial Connexin and Pannexin Functions in Rodent Models of Nervous System Diseases

**DOI:** 10.3389/fncel.2019.00491

**Published:** 2019-11-06

**Authors:** Lucila Brocardo, Luis Ernesto Acosta, Ana Paula Piantanida, Lorena Rela

**Affiliations:** Grupo de Neurociencia de Sistemas, Facultad de Medicina, Instituto de Fisiología y Biofísica Bernardo Houssay (IFIBIO Houssay), Consejo Nacional de Investigaciones Científicas y Técnicas (CONICET), Universidad de Buenos Aires, Buenos Aires, Argentina

**Keywords:** connexins, pannexins, gap junctions, hemichannels, astrocytes, microglia, plasticity

## Abstract

A variety of glial cell functions are supported by connexin and pannexin proteins. These functions include the modulation of synaptic gain, the control of excitability through regulation of the ion and neurotransmitter composition of the extracellular milieu and the promotion of neuronal survival. Connexins and pannexins support these functions through diverse molecular mechanisms, including channel and non-channel functions. The former comprise the formation of gap junction-mediated networks supported by connexin intercellular channels and the formation of pore-like membrane structures or hemichannels formed by both connexins and pannexins. Non-channel functions involve adhesion properties and the participation in signaling intracellular cascades. Pathological conditions of the nervous system such as ischemia, neurodegeneration, pathogen infection, trauma and tumors are characterized by distinctive remodeling of connexin expression and function. However, whether these changes can be interpreted as part of the pathogenesis, or as beneficial compensatory effects, remains under debate. Here we review the available evidence addressing this matter with a special emphasis in mouse models with selective manipulation of glial connexin and pannexin proteins *in vivo*. We postulate that the beneficial vs. detrimental effects of glial connexin remodeling in pathological conditions depend on the impact of remodeling on the different connexin and pannexin channel and non-channel functions, on the characteristics of the inflammatory environment and on the type of interaction among glial cells types.

## Introduction

Connexins are transmembrane proteins with a variety of physiological roles including channel and non-channel functions. Channel functions involve the formation of gap junctions, which are intercellular connections, permeable to ions and small metabolites, formed by the apposition of connexin hexamers (connexons) provided by each of the participating cells. Gap junction-mediated glial networks are ubiquitous in the central nervous system and involve astrocytes and oligodendrocytes. The type of connexins involved in the interaction depends on the cell types participating in the connection (Rouach et al., 2002; Giaume and Theis, 2010; Takeuchi and Suzumura, 2014; Abudara et al., 2018). Connexins can also function as membrane hemichannels with the potential to release mediators such as ATP and glutamate from astrocytes and microglia (Orellana et al., 2013; Gajardo-Gómez et al., 2016; Nielsen et al., 2017). Non-channel functions of connexins involve adhesion properties and intracellular cascade signaling (Zhou and Jiang, 2014; Leithe et al., 2018).

The ubiquitous nature and diversity of connexin functional properties make them versatile mediators of glial cell physiology and a putative locus for maladaptive plasticity in pathological conditions. Plastic changes in abundance and/or functions of glial connexins have been reported in a variety of pathology models (Belousov et al., 2017; De Bock et al., 2017; Xing et al., 2019). However, whether connexin plasticity is causal to the pathology, or is an epiphenomenon of pathological conditions, is becoming to be elucidated only recently, owing to the availability of tools for selective manipulation of glial connexins *in vivo* (Giaume and Theis, 2010). Here we review the available work using rodent models with glia-selective connexin manipulations to evaluate causal relations between glial connexin plasticity and neuropathology, focusing on traumatic and neurodegenerative diseases, ischemia and brain tumors. Most of the cases reviewed here will focus on connexin 43 (Cx43), which is by far the most studied glial connexin and dominates the available literature. We will also review the scarce literature available on glia-selective pannexin manipulations in these models, and discuss the glia-selective data in the context of additionally more abundant data using global connexin and pannexin manipulations.

Modulation of connexin expression and function that may emerge in the context of hypoxia, inflammation and injury, both *in vitro* and *in vivo*, have been thoroughly reviewed and putative mechanisms underlying the observed changes have been proposed (Rouach et al., 2002; Contreras et al., 2004; Farahani et al., 2005; Kielian, 2008; Orellana et al., 2009; Eugenin et al., 2012; Koulakoff et al., 2012; Quintanilla et al., 2012; Bosch and Kielian, 2014; Freitas-Andrade and Naus, 2016). A few important points emerging from the body of available literature are worth mentioning before we focus on glia-selective *in vivo* manipulations, which is the focus of this review. The first point is that a change in connexin immunolabeling needs to be interpreted with caution. Connexin channels in the context of gap junction plaques or in a disassembled state can have dramatically different immunoreactive properties, thus connexins may appear absent owing to epitope masking (Theriault et al., 1997). This highlights the importance of using independent approaches to measure connexin abundance and function. A second point is that changes in the expression of glial connexins in pathological conditions are connexin-, time-, region- and model-specific. As an example, Borna virus infection of neonatal rats results in neurodegeneration and widespread reactivity of hippocampal astrocytes when analyzed 2 months after infection (Köster-Patzlaff et al., 2007). This astrocytic activation is accompanied by an increase of Cx43 in all layers of the dentate gyrus of the hippocampus and a decrease of the same connexin in the CA3 pyramidal layer. Another typically astrocytic connexin, connexin 30 (Cx30), increased in all the hippocampal regions mentioned above (Köster-Patzlaff et al., 2007). In contrast, in a mouse model proposed to mimic features of aging and Alzheimer’s disease induced by ovariectomy and chronic D-galactose administration, a reduction of Cx43 in the dentate gyrus and CA1 regions of the hippocampus was observed, while no change was observed in the CA3 hippocampal region (Liu et al., 2010). This was suggested to reflect differential resistance to neuronal damage of CA3 compared to CA1, as was observed in a model of ischemia in rats (Rami et al., 2001). Thus, generalizations about connexin plasticity across brain regions and pathologies should be cautious. A third issue is that changes in the abundance of glial connexin proteins in pathological conditions is challenging to interpret without parallel assessments of functional studies of connexin functions, such as the extent of gap junction coupling, the permeability of connexin hemichannels available at the membrane surface (Sáez et al., 2013) and the phosphorylation state of specific connexin sites that can mediate intracellular signaling. These functions may be oppositely modulated and/or have opposing effects on the severity of the pathology (Retamal et al., 2007; Karpuk et al., 2011). Therefore, when manipulations target more than one connexin function—e.g., both gap junctions and hemichannels—this has to be clearly stated and the effects of the manipulation should be interpreted with care. Finally, an important idea, emerging mostly from *in vitro* studies is that different connexins are expressed in different cell types of the nervous system—i.e., neurons, astrocytes, oligodendrocytes and microglia—and may interact in complex ways when modulated in pathologic conditions. Even more, glial pannexin proteins, which form membrane channels showing structure and function similarity to connexin hemichannels, can also be modulated in inflammatory conditions and have been proposed to play a role in pathologic conditions (Freitas-Andrade and Naus, 2016; Lapato and Tiwari-Woodruff, 2018). The contribution of Cx43 and pannexin-1 (Px1) to hemichannel activity in astrocytes has been a matter of debate as different studies assigned a preponderant role to one or the other, depending on the conditions analyzed (Iglesias et al., 2009; Orellana et al., 2011). Hence, cell type-selective manipulations of connexins and pannexins are essential to achieve a comprehensive idea of their roles in pathology. With these general ideas in mind, we will review the available literature that aims at using *in vivo* selective manipulations of glial connexin and pannexin functions to address their roles in a subset of pathologic conditions.

For this purpose, we used keyword search in PubMed and Scopus citation databases using combinations of keywords (Table 1). We then reviewed the methods section of all articles yielded by each search and selected those articles that used *in vivo* rodent models. A final selection of work using global and cell-specific connexin and pannexin manipulations were selected for detailed analysis.

**Table 1:** Number of original research articles found for the keyword combinations used for the literature search in PubMed and Scopus citation databases.

	Connexin	Pannexin
Astrocyte	875	83
Microglia	150	39
Oligodendrocyte	212	5
Ependymal	28	1
Endothelial + Brain	93	6
Tumor + Brain	212	19

## Neurodegenerative Diseases

We mentioned that connexins are expressed in different glial types and may interact in complex ways. To illustrate these complex interactions, we can mention a study by Orellana et al. (2011) that addressed the involvement of glial connexins and pannexins in neuronal death associated with exposure to a toxic fragment of the amyloid precursor protein, whose accumulation is associated with Alzheimer’s disease. Orellana et al. (2011) observed that in cultured cortical astrocytes and microglia, toxic amyloid fragments increased hemichannel activity. While astrocyte hemichannel activity was explained mainly by Cx43 availability, microglial hemichannel activity involved both Cx43 and Px1 (Orellana et al., 2011). This amyloid-induced glial hemichannel activity was associated with the release of metabolites that are toxic for neurons, likely ATP and glutamate. While microglia-mediated neurotoxicity could be prevented by combined application of Px1 and Cx43 blockers, astrocyte-mediated neurotoxicity was abolished by Cx43 block alone (Orellana et al., 2011). In this same study, hippocampal slices in organotypic culture exposed to the toxic amyloid fragment showed increased hemichannel activity. This effect was revealed by dye uptake first in microglia, then in astrocytes and finally in neurons. While Cx43 blockers and microglia inactivation with minocycline completely prevented amyloid-induced neuronal death, in hippocampal slices obtained from mice with astrocyte-selective deficiency of Cx43 there was only a partial protection (Orellana et al., 2011). These data suggest that astrocytic recruitment is downstream microglial activation, however, pure astrocyte cultures can produce neurotoxic conditioned medium in the presence of toxic amyloid fragments. The relative contributions of microglia and astrocyte connexins to amyloid-induced neurotoxicity will need microglia-selective manipulations (Parkhurst et al., 2013; Yona et al., 2013; Zhao et al., 2019) of connexin and/or pannexin proteins for further clarification.

It is important to note that amyloid neurotoxicity modeled in cell and organotypic slice culture systems involve a rather homogeneous exposure to the toxic fragment. This homogeneous exposure does not reproduce the neurotoxicity gradients generated in the pathology characterized by amyloid deposition in the form of amyloid plaques. The relevance of these gradients for glial plasticity in Alzheimer’s disease was nicely illustrated in a mouse model of familial Alzheimer’s disease (APP_swe_/PS1_dE9_ mice, or APP/PS1 mice) characterized by aging-dependent plaque formation. Hemichannel activity assessed using dye uptake in acute brain slices was enhanced in hippocampal astrocytes of APP/PS1 mice when compared to control mice. Interestingly, hemichannel hyperactivity in APP/PS1 astrocytes involved Cx43 and Px1 when astrocytes were located near amyloid plaques, while the increased hemichannel activity observed in astrocytes far from plaques involved only Cx43 (Yi et al., 2016). Microglial inhibition by minocycline partially reduced hemichannel hyperactivity in astrocytes near plaques, while astrocytes far from plaques remained unaffected. When the effects of the APP/PS1 mutations on hemichannel activity were analyzed in the context of a selective deficiency of Cx43 in astrocytes, almost no hemichannel activity was observed in astrocytes far from plaques, but a Px1 component of hemichannel activity remained in astrocytes near plaques. Thus, Px1 appears to contribute to astrocyte hemichannel activity in some *in vivo* conditions (Yi et al., 2016). Together with the prevention of hemichannel activity in astrocytes, the astrocyte-selective Cx43 deficiency in APP/PS1 mice was associated with less mitochondrial oxidative stress assessed by MitoSOX superoxide indicator and less dystrophic dendrites, assessed by reticulon 3 (RTN3) immunoreactivity (Yi et al., 2016), indicating that astrocyte Cx43 worsened the neurotoxicity of the environment. Of interest, no differences were observed in indicators of astrocytic gap junction coupling between APP/PS1 and control mice, assessed by fluorescence recovery after photobleaching (FRAP; Yi et al., 2016), strengthening the idea that the neurotoxic effect of astrocytes was exerted *via* Cx43 hemichannels. Further, APP/PS1 mice with astrocyte Cx43 deficiency had dramatically reduced astrogliosis and improved cognitive performance compared with APP/PS1 mice. Even more, re-expression of Cx43 in Cx43-deficient APP/PS1 mice using an astrocyte-directed adeno-associated viral vector reinstated the cognitive impairment (Ren et al., 2018). These results indicate that in the sequence of events of microglial and astrocytic activation that were proposed to lead to neuronal damage in Alzheimer’s disease based on *in vitro* studies, the increase of Cx43 hemichannel activity in astrocytes is a deleterious process and this is confirmed in an *in vivo* model that selectively manipulates connexins in astrocytes.

Evidence for the idea that connexins participate in the complex interaction between astrocytes and microglia during neuroinflammation was provided as well by studies done in experimental autoimmune encephalomyelitis, a mouse model of multiple sclerosis. In this model, the pathologic condition is produced by subcutaneous immunization with myelin oligodendrocyte glycoprotein MOG_35–55_ peptide and intraperitoneal administration of Pertussis toxin on the day of immunization and 2 days after (Chen and Brosnan, 2006). This model is characterized by progressive paralysis followed by partial remission after an acute peak of clinical signs, associated with inflammatory infiltrate, demyelination and axonal damage (Chen and Brosnan, 2006). Mice with a selective astrocytic Cx43 deficiency or with this deficiency in a global Cx30 knockout background to prevent compensatory effects were not statistically different from wild type mice in terms of a clinical score that evaluates the severity and time course of the motor signs of experimental autoimmune encephalomyelitis up to 3–4 weeks after immunization (Lutz et al., 2012). These mice were not different either in terms of the degree of immune cell reactivity in the spinal cord, evaluated using ionized calcium binding adaptor molecule 1 (Iba1) immunostaining at the endpoint of clinical evaluation. Interestingly, the global Cx30 deficiency reduced the severity of the chronic phase (8 weeks after the insult) in this model and this effect was correlated with the emergence of an anti-inflammatory phenotype in both spinal cord astrocytes and microglia (Fang et al., 2018). Given that a reduction in the abundance of Cx43 during the chronic phase of experimental autoimmune encephalomyelitis was observed in global Cx30 deficient mice, the neuroprotective effect could be mediated indirectly *via* astrocytic Cx43. This possibility will need to be specifically tested ideally using conditional astrocytic-selective and inducible Cx43 deficiency alone or in combination with Cx30 deficiency. Furthermore, global Px1 deficiency delayed the onset of symptoms in this model of experimental autoimmune encephalomyelitis, improving the clinical state of mice in the acute phase. However, Px1-deficient mice reached a clinical score that was comparable to the one observed in wild type mice during the chronic phase (Lutz et al., 2013). When ATP release was evaluated in the bathing medium of spinal cord slices, the authors observed that the enhancement of ATP release that the insult produced was significantly smaller in slices obtained from Px1-deficient mice when compared to wild type slices (Lutz et al., 2013). The former results are compatible with a sequential involvement of Px1 and Cx30 -and possibly Cx43- in the progression of experimental autoimmune encephalomyelitis. Further experiments using glial cell type-selective pannexin and connexin manipulations will help to determine whether an early microglia dependent process involving Px1 elicits a late astrocyte-dependent process involving connexins as part of the pathogenesis of experimental autoimmune encephalomyelitis.

The importance of connexin and pannexin functions to explain pathological conditions characterized by neuronal dysfunction and degeneration is highlighted by data coming from studies of human diseases linked to connexin and pannexin mutations. X-linked Charcot-Marie-Tooth disease type 1 (OMIM #302800) is characterized by progressive muscle weakness and atrophy associated with mutations in the *GJB1* gene, coding for connexin Cx32 (Cx32). These mutations are linked to peripheral demyelination and axonal degeneration that can be accompanied by central manifestations (Wang and Yin, 2016). Deletions of the *GJB1* coding sequence in humans produce a predominantly peripheral phenotype that is compatible with the alterations observed in Cx32 null mice (Hahn et al., 2000; Nakagawa et al., 2001). The central manifestations can be transient, do not appear to correlate with the severity of peripheral neuropathy and may be triggered by a variety of stress stimuli (Wang and Yin, 2016). Besides peripheral signs of neuropathy, Cx32 knockout mice show increased signs of widespread central nervous system inflammation when compared to wild type mice, both in basal conditions and after a systemic challenge with lipopolysaccharide (Olympiou et al., 2016). Interestingly, Cx32 knockout mice bearing a human Cx32 mutation associated with central manifestations of X-linked Charcot-Marie-Tooth disease (Cx32KOT55I) display an enhanced widespread central nervous system inflammatory response not associated with oligodendrocyte loss after lipopolysaccharide challenge, when compared with Cx32 knockout mice that do not carry this mutation (Olympiou et al., 2016). These data suggest that Cx32 mutations contribute to the disease not only through loss-of-function mechanisms that promote central neuroinflammation in addition to peripheral neuropathy, but also through gain-of-function mechanisms that increase the susceptibility to inflammatory challenges. The signals underlying the enhanced neuroinflammation remain to be elucidated.

Another demyelinating disease associated with mutations in the *GJC2* gene coding for connexin 47 (Cx47) is Pelizaeus-Merzbacher-like disease type 1 (OMIM #608804), characterized by progressive spasticity and ataxia. When compared with wild type mice, both knockout mice for Cx47 and mice carrying a mutation associated with the disease in humans (Cx47KOM282T) show delayed myelination and mild motor impairment during the juvenile stage, together with cerebellar astro- and microgliosis, that is no longer observable in the adult stage owing to compensation by Cx32 (Tress et al., 2011). As is the case for Cx32-deficient and mutant mice, Cx47-deficient and mutant mice show evidence of gap junction disassembly, not only in relation to oligodendrocyte-oligodendrocyte connections but also for oligodendrocyte-astrocyte connections (Tress et al., 2011; Olympiou et al., 2016). Whether augmented signs of neuroinflammation in these connexin-deficient mice is associated with oligodendrocytic, astrocytic or neuronal dysfunction, or with a combination of signals from these cell types, remains to be established.

## Ischemia

The question whether astrocytic connexins are beneficial or detrimental in pathological conditions fueled a rich debate and is well illustrated for the case of ischemia (Giaume et al., 2007). Correlational evidence and nonselective manipulations aiming at inhibition of Cx43 function in astrocytes led to the idea that Cx43 contributed to the amplification of ischemic damage, as the treatment with octanol, a nonselective connexin and gap junction blocker, reduced the infarct size in rat models of cerebral ischemia by carotid or middle cerebral artery occlusion (Rawanduzy et al., 1997; Rami et al., 2001).

Contrasting with the former data, in a mouse model of ischemia produced by middle cerebral artery occlusion during 45 min with evaluation 24 h later, the infarct size was larger in mice with a selective Cx43 astrocytic deficiency when compared to wild type mice (Lin et al., 2008). In addition, an experience of hypoxic preconditioning of 5 h at 8% oxygen 3 days prior to artery occlusion produced an approximate 50% reduction in infarct size in wild type mice, and was absent in mice with astrocytic Cx43 deficiency (Lin et al., 2008). The protective effect of hypoxic preconditioning needed the availability of adenosine A1 receptors and it was proposed to be produced by ATP release by Cx43 hemichannels and subsequent metabolization to adenosine extracellularly (Lin et al., 2008). Given that astrocytic Cx43 deficiency is partly compensated by an increase in Cx30 expression, the mice used in these experiments were also global Cx30 knockouts (Wallraff et al., 2006). Cx43 was proposed as pivotal for neuroprotection in this cerebral ischemia model and was reinforced by consistent results obtained in mice with conditional deletion of Cx43 in astrocytes and no deletion of Cx30 (Nakase et al., 2003). In these mice, more cell death and enhanced number of inflammatory cells were observed in the area of penumbra without significant changes in the volume affected by astrogliosis, when compared with control mice (Nakase et al., 2004). The protective effect of Cx43 against ischemic damage was further reinforced by another study using a mouse line bearing a Cx43 mutation that affects Cx43-mediated gap junction and hemichannel activity (Gja1^Jrt^; Kozoriz et al., 2013). Thus, in contrast to what was observed for the inflammatory environment of Alzheimer’s disease models, Cx43 was regarded as neuroprotective under hypoxic conditions. One important difference called into attention to explain the difference between these two conditions is the chronicity of the insult in Alzheimer’s disease models compared to the acute insult in the models of ischemia.

Genetic models that allow researchers to restrict the connexin deficiency to specific glial types were a big step forward to address glial connexin functions in nervous system pathology; however, there are a series of confounding effects that the strategy does not address. First, most of the data come from constitutive lines that tie the connexin deletion of interest to the timing of expression of a particular promoter, such as the promoter for glial fibrillary acidic protein (GFAP), which is also expressed by neural progenitor cells (Giaume and Theis, 2010). This caveat can be minimized with new tools that spare neural progenitor cells (Tsai et al., 2012; García-Cáceres et al., 2016; Srinivasan et al., 2016; Koh et al., 2017) or inducible tools that allow the researchers to evaluate phenomena in time windows shorter than the time needed for generation of new neurons (Burns et al., 2007; Rivers et al., 2008). Second, the deletion of connexin coding sequences eliminates all connexin functions, thus, it cannot address hemichannel, gap junction and non-channel connexin functions separately. This is particularly relevant in light of data showing that the permeability of gap junction channels and hemichannels can be oppositely modulated in an *in vitro* model of injury in which hemichannels were activated and gap junction coupling was reduced (Retamal et al., 2007). A promising tool to dissociate Cx43 hemichannel and gap junction channel functions is the connexin mimetic peptide Gap19, which blocks Cx43 hemichannels within the hour after exposure without affecting Cx43-mediated gap junction coupling. At larger exposure times, it slightly enhances gap junction coupling, so it nicely dissociates the two functions (Wang et al., 2013). A recent study assessed the effects of this peptide in male mice subjected to a cerebral ischemia-reperfusion protocol (45 min of middle cerebral artery occlusion and assessment 24 h after initiation of reperfusion). Intracerebroventricular administration of Gap19 30 min before ischemia had a protective effect, significantly reducing infarct volume (Chen et al., 2019). This result indicates that Cx43 hemichannel function is deleterious in ischemia and suggests that the increase in infarct volume observed in mice with astrocytic Cx43 deficiency (Nakase et al., 2004) obeys to a neuroprotective role of Cx43-mediated gap junction channels that overrides the neurotoxic action of hemichannels. However, as Gap19 also downregulated Cx43 expression by about 30% and astrocytic gap junction connectivity was not assessed (Chen et al., 2019), a contribution of astrocytic gap junctions to explain the neuroprotective effect of Gap19 cannot be ruled out. Potential effects of Gap19 in microglia or other targets could also explain the results, as the manipulation was not cell-type selective. Further support for the idea that an increment of astrocytic Cx43 hemichannel activity in response to ischemic insults is detrimental comes from a study using mice with global site-directed mutagenesis to eliminate functional phosphorylation sites that are a substrate for mitogen-activated protein kinase in the C-terminus of Cx43 (Freitas-Andrade et al., 2019). These mice (MK4 mice) show reduced infarct size after permanent middle cerebral artery occlusion when compared with wild type mice and two other mouse lines targeting phosphorylation sites for protein kinase C and caseine kinase 1, these two displaying no protection. Cultured astrocytes from MK4 mice showed reduced hemichannel activity assessed using dye uptake and electrophysiological measurements. In addition, MK4 astrocyte gap junction coupling assessed through a scrape-loading assay in cortical brain slices was indistinguishably increased in MK4 and wild type mice after the ischemic insult (Freitas-Andrade et al., 2019). Given that this was a global manipulation, it remains to be confirmed that the protective effect is astrocytic Cx43-dependent.

Non-channel functions of Cx43 involve adhesion properties and the participation in intracellular signaling *via* the C-terminal domain. Cx43 is codified by the *Gja1* gene, which can produce alternative carboxyl-terminal fragments *via* translation at alternative initiation sites (Ul-Hussain et al., 2014). Among these fragments, the GJA1-20k variant is selectively upregulated in a rat model of brain hypoxia-ischemia and may explain at least part of the neuroprotective effects produced by Cx43 in hypoxic conditions. Mice with a C-terminal-truncation in the Cx43 coding region show more vulnerability to brain ischemia, however, as astrocytic gap junction and hemichannel functions are partially disrupted as well in these mice, a neuroprotective role of the carboxyl-terminal fragment of Cx43, independent of its impact in channel functions, cannot be confirmed (Kozoriz et al., 2010).

Multiple connexins and glial types likely contribute to the outcome after ischemic damage. This idea is reinforced by the observation that male null mice for Cx32 are more vulnerable to global cerebral ischemia produced by transient (10 min) bilateral common carotid artery occlusion (Oguro et al., 2001). This enhanced vulnerability was observed 7 days after the insult as increased neuronal loss in the CA1 hippocampal region of null mice when compared with wild type mice (Oguro et al., 2001). Given that Cx32 is expressed by oligodendrocytes and by parvalbumin-positive inhibitory interneurons in the hippocampus, the locus of origin of the Cx32-dependent neuroprotective effect could not be elucidated in this model, highlighting the need of glia type-selective manipulations of connexin proteins to address their functions (Doerflinger et al., 2003; Piantanida et al., 2019).

The contribution of pannexin proteins to the outcome after ischemic brain damage has been addressed using global knockout mice for Px1 and pannexin-2 (Px2), two pannexin proteins expressed in the nervous system. Contrasting with the deleterious effects of global Cx32 deficiency and astrocytic Cx43 deficiency, global combined deficiency of Px1 and Px2 results in protection against ischemic damage when evaluated 48 h after permanent middle cerebral artery occlusion (Bargiotas et al., 2011, 2012). Interestingly, astrocytes in primary culture with combined Px1 and Px2 deficiency show normal ATP release and membrane conductance, indicating that pannexins do not contribute significantly to astrocytic channel function in these *in vitro* conditions. In contrast, Px1- and Px2-deficient cortical neurons in primary culture show defective dye release (Bargiotas et al., 2011). These results suggest that pannexins contribute to ischemic damage expansion through a neuron-dependent mechanism. This is supported by a study showing comparable degrees of protection against ischemia-reperfusion damage in retinas of global and neuron-selective Px1 deficiency (Dvoriantchikova et al., 2012). Interestingly, microglial cells of Px1 deficient mice retain their ability to display rapid morphologic plasticity in response to extracellular ATP and other stimuli associated with injury (Dissing-Olesen et al., 2014). However, a possible contribution of microglia or endothelial cells (Gaynullina et al., 2014; Sharma et al., 2018) to explain the neuroprotective effect of global Px1 and Px2 deletion cannot be ruled out and needs to be specifically tested.

The availability of new mouse lines to achieve conditional deletion of Px1 using Cre-lox technology (Dvoriantchikova et al., 2012) and conditional expression of tamoxifen-inducible Cre recombinase in microglia and leptomeninges, sparing blood monocytes (Kaiser and Feng, 2019) is much promising. Given that Px2 can compensate for Px1 deficiency, and vice versa (Bargiotas et al., 2011) an *in vivo* approach to address the impact of microglia-selective Px1 deficiency on ischemic damage and other pathologic conditions will likely need to combine the conditional microglia-selective Px1 deletion with a global (or conditional, when available) Px2 deletion. This is an analogous strategy to the one used to address the roles of astrocytic Cx43 without compensation by Cx30 (Wallraff et al., 2006). One additional factor that has to be taken into consideration is the observation of sexual dimorphism in the neuroprotective effect of global Px1 deficiency. When evaluated 48 h after middle cerebral artery occlusion without sex discrimination, global Px1 deficiency did not show protection against ischemic damage (Bargiotas et al., 2011). Of note, another study using a middle cerebral artery occlusion lesion model in an independently generated Px1-knockout line observed that females with Px1 deficiency showed approximately 50% smaller size infarcts 4 days after the lesion when compared with wild type females (Freitas-Andrade et al., 2017). In contrast, males with Px1 deficiency displayed lesion sizes that were indistinguishable from lesions in wild type male mice. The differential susceptibility to ischemic damage between males and females correlated with differential astrocytic reactivity and degree of neuroinflamation, assessed through GFAP and Iba1 immunostaining, respectively, which were less dramatic in Px1-deficient female mice when compared to wild type female mice and males of either genotype (Freitas-Andrade et al., 2017). These data highlight the need to analyze the possibility for sexually dimorphic responses to nervous system insults before concluding a lack of effect of manipulations.

Overall, the current available data strongly suggest that therapeutic interventions to prevent ischemic damage will benefit from strengthening the efforts to develop tools to counteract connexin and pannexin hemichannel activity while preserving connexin gap junction function.

## Spinal Cord Injury

Female mice with selective astrocytic Cx43 deficiency in a Cx30-knockout background were also assessed to evaluate their response to spinal cord contusion injury, and compared to controls that had only the global Cx30 deficiency (Huang et al., 2012). This study reported that the response to weight-drop spinal cord injury involved less ATP release in the peritraumatic area 1 h after injury in mice with astrocytic Cx43 deficiency. These mice also showed reduced astrocytic and microglial reactivity 1 week after injury and smaller lesion sizes than control mice when evaluated 8 weeks after injury, accompanied by an improved functional recovery that was observed as early as 3 days after damage (Huang et al., 2012). Thus, in this injury model, Cx43 availability was deleterious. In another model of spinal cord contusion injury in rats, peptide5, a Cx43 mimetic peptide that blocks Cx43 hemichannels and gap junctions (O’Carroll et al., 2008), administered at the lesion site 1 h after the lesion, diminished astrocytic activation and inflammatory cell numbers at the lesion site, promoted neuronal survival, and improved locomotor recovery when compared with the administration of a control peptide (O’Carroll et al., 2013). These data are consistent with the more selective astrocyte-directed manipulation of Cx43 mentioned above and support the idea that Cx43 is deleterious for the outcome of traumatic spinal cord injury. Interestingly, comparable results were obtained when peptide5 was administered systemically to rats with spinal cord contusion (Mao et al., 2017b). Consistent results were obtained in yet another study using female mice treated systemically with INI-0602, a derivative of the broadly used non-selective connexin and pannexin blocker carbenoxolone (Bruzzone et al., 2005; Dahl et al., 2013), that prevents the release of glutamate from mouse cultured microglia in response to lipopolysaccharide administration (Takeuchi et al., 2011). Administration of INI-0602, starting at the time of injury and during a month, improved the outcome of transection spinal cord injury, reducing astrogliosis and inflammation, and improving motor recovery (Umebayashi et al., 2014). These results emphasize the idea that even when cell-type selective manipulations are essential to address the mechanisms behind the resolution of injuries, non-selective manipulations have potential as useful treatments, provided that there are no significant undesired off-targets for the therapeutic agent (Mao et al., 2017a).

In contrast to what was reported in spinal cord injury models, mice with astrocytic Cx43 deletion in a wild type Cx30 background increased the extent of astrogliosis and inflammatory cells around the lesioned area in a brain stab wound injury model (Theodoric et al., 2012). A number of factors may explain these differences, such as the type of injury, the lesion site, the time of observation after injury and the cellular microenvironment around the wound.

## Neuropathic Pain

The development of neuropathic pain after peripheral or central damage involves plastic changes in spinal nociceptive circuits that involve glial plasticity as well. Selective manipulation of astrocytes and microglia have recently revealed that connexin and pannexin proteins in these glial types are critical for the development of neuropathic pain. In a mouse model of mild spinal cord injury, selective astrocytic Cx43 deficiency in a Cx30 knockout background reduced the development of mechanical allodynia and heat hyperalgesia, when compared with Cx30 knockout and wild type mice (Chen et al., 2012). On the other hand, Px1 deficiency in myeloid cells including microglia attenuated the development of mechanical allodynia in a model of induced osteoarthritis by intra-articular administration of monosodium iodoacetate. Interestingly, the hyperactivity of spinal dorsal horn neurons associated with the development of mechanical allodynia was also attenuated by myeloid cell-selective deletion of Px1 (Mousseau et al., 2018). It is important to note that this study used an inducible knock-in/knock-out Cre-lox system that allowed the researchers to evaluate the acute effects of eliminating Px1 expression in the myeloid lineage. Another study used a model in which mechanical allodynia also develops after spared nerve injury of the sciatic nerve. This study did not observe a reduction of pain thresholds in mice with Px1 deficiency in the myeloid lineage using a non-inducible transgenic Cre-lox system under comparable regulatory sequences (the Cx3cr1 promoter; Weaver et al., 2017). In contrast, this same study did observe a prevention of nerve injury-induced mechanical allodynia in global Px1-deficient mice. These results suggest that the non-inducible deletion of Px1 from myeloid cells can be developmentally compensated, while the global Px1 deletion cannot. Strikingly, Weaver et al. could reinstate the capability for nerve injury-induced mechanical allodynia in global Px1-deficient mice after wild type bone marrow transplantation, indicating that bone marrow-derived immune cells confer at least part of the Px1 dependence of nerve injury-induced mechanical allodynia. Combined manipulations of astrocytic connexins and microglial pannexins in specific time windows will help elucidate whether and how these cell types and molecules interact for the development of neuropathic pain. The availability of tools to genetically manipulate astrocytes and microglia with different inductor drugs (Tanaka et al., 2012; Srinivasan et al., 2016) is a promising new avenue with potential to assess interactions between these cell types. The generation of new tools designed to achieve targeted deletions of a broader diversity of connexins and pannexins than what is available (Liao et al., 2001; Cohen-Salmon et al., 2002; Poon et al., 2014; Clasadonte et al., 2017) will overcome one of the current limitations.

## Tumors

Besides their participation in the resolution of nervous system damage, astrocytes are determinants of the microenvironment in which brain tumors develop. In addition, tumor cells express connexins and there is evidence using dye transfer *in vitro* and *in vivo* that they can establish connexin-mediated gap junctions with non-tumor cells (Zhang et al., 1999, 2003). However, a direct electrophysiological measurement of gap junction coupling between astrocytes and tumor cells is still lacking. Data supporting the relevance of connexin proteins as potential targets for antitumor therapy have been reviewed recently (Aasen et al., 2017; Umans and Sontheimer, 2018; Uzu et al., 2018). Whether connexin gap junction, hemichannel and/or non-channel functions are important for tumor progression has not been resolved. However, there is compelling evidence that some tumor cells downregulate connexin proteins and that restoring connexin expression reduces malignancy. As an example, C6 rat glioma cells express low levels of Cx43 and Cx30. When implanted intracranially, they produce well-defined large tumors in the brain parenchyma, resulting in a survival rate of 43% of transplanted individuals after 30 days of implantation. However, rats implanted with Cx30 cDNA-transfected C6 glioma cells exhibited 66% of survival rate, and smaller tumor masses were detected by MRI analysis (Arun et al., 2017). Interestingly, Px1-transfected C6 glioma cells in culture showed reduced proliferation and motility and displayed dye coupling, which was virtually absent in untransfected C6 cells (Lai et al., 2007). When inoculated to nude mice, Px1-transfected C6 glioma cells produced significantly smaller tumors than cells transfected with a control construct. Similar results were observed for Px2-transfected C6 glioma cells (Lai et al., 2009).

Contrasting with the former results, in a brain metastasis model using nude mice inoculated with mammary adenocarcinoma cells of human origin *via* the left cardiac ventricle, shRNA-mediated depletion of Cx43 from cancerous cells reduced metastatic growth in the brain parenchyma when assessed 2 weeks after inoculation (Chen et al., 2016, 2017). Wild-type Cx43 re-expression in Cx43-depleted cancerous cells rescued metastatic activity, whereas re-expression of a mutated variant of Cx43 that does not form functional channels [Cx43(T154A)] did not (Beahm et al., 2006). Interestingly, Cx43 promoted proliferation of metastatic cells in the brain, but not extravasation across the blood-brain barrier, as Cx43 depletion in cancer cells did not significantly diminish the number of cancer cells in the brain parenchyma, but micrometastases showed decreased proliferative activity when assessed 1 week after cancer cell inoculation. Based on a large body of accompanying *in vitro* data, the authors proposed a model in which gap junctions between tumor cells and astrocytes are required for cancer cell growth and survival (Chen et al., 2016). However, a potential hemichannel function of Cx43 in this process cannot be discarded.

Further research with *in vivo* models and tumor cell manipulations targeting specific connexin functions will shed light on the roles of connexins expressed by cancer cells for their capacity to invade the brain parenchyma, their proliferation and survival, which may differ depending on cancer cell type. To conclude, we will focus on the analysis of plasticity of astrocyte connexin functions in the proximity of tumor cells and their possible detrimental or beneficial effects for tumor progression.

In mice with Cx43-deficient astrocytes, brain implantation of mouse GL261 glioma cells resulted in a larger extent of astrogliosis near the tumor implant, together with reduced invasion of tumor cells into the brain parenchyma. This result was replicated when Cx43-knockdown glioma cells were implanted in wild type mice (Sin et al., 2016). To determine which Cx43 function was taking part in this process, glioma cells were transfected with cDNA coding for the channel-defective Cx43 mutant variant, Cx43(T154A), and implanted in astrocyte-selective Cx43-deficient mice, or control mice. Tumor invasion was compromised only in the astrocyte Cx43 null background, suggesting that heterocellular (glioma-astrocyte) gap junction channel activity is not necessary for tumor invasion. However, release of astrocyte gliotransmitters *via* Cx43 hemichannels cannot be discarded. In addition, the outcome of implanted glioma cells was assessed in Cx43^ΔCT/−^ mice carrying one astrocyte-selective null Cx43 allele and one ΔCT allele that consists in a truncated Cx43 variant (Cx43K258stop). This variant lacks a cytoplasmic tail that carries phosphorylation sites and protein-interacting domains (Freitas-Andrade et al., 2019) and exhibits reduced gap junction activity and increased hemichannel activity when assessed in cultured astrocytes using dye transfer and uptake techniques (Sin et al., 2016). Cx43^ΔCT/−^ mice displayed compromised invasion of tumor cells in the brain parenchyma and increased astrogliosis when compared with Cx43^+/–^ (mice carrying one astrocyte-selective Cx43 null allele and one wild type allele; Sin et al., 2016). These results indicate that astrocytic Cx43 constitutes a means for tumor invasion in the brain. The channel and non-channel functions directly involved in tumor invasion and survival are still elusive; however, at least in the former case, the results seem to discard adhesive properties of the extracellular loops, which remained intact in the mutant variants used, provided that Cx43 expression levels and traffic to the membrane remained invariant.

## Concluding Remarks and Future Directions

**Figure 1:**
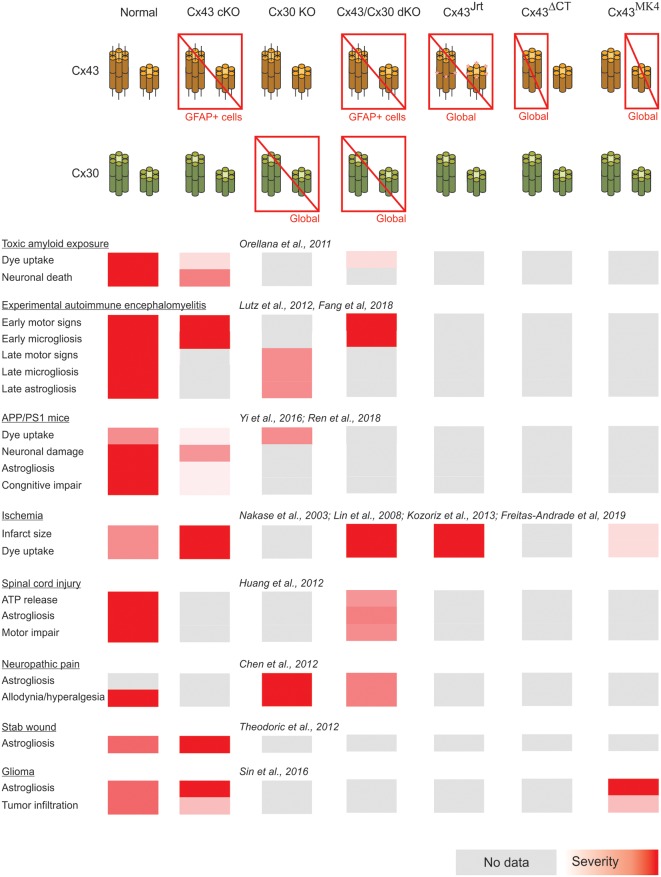
Summary of the impact of global and astrocyte-selective connexin function disruption in the context of *in vivo* models of central nervous system disease.

**Figure 2:**
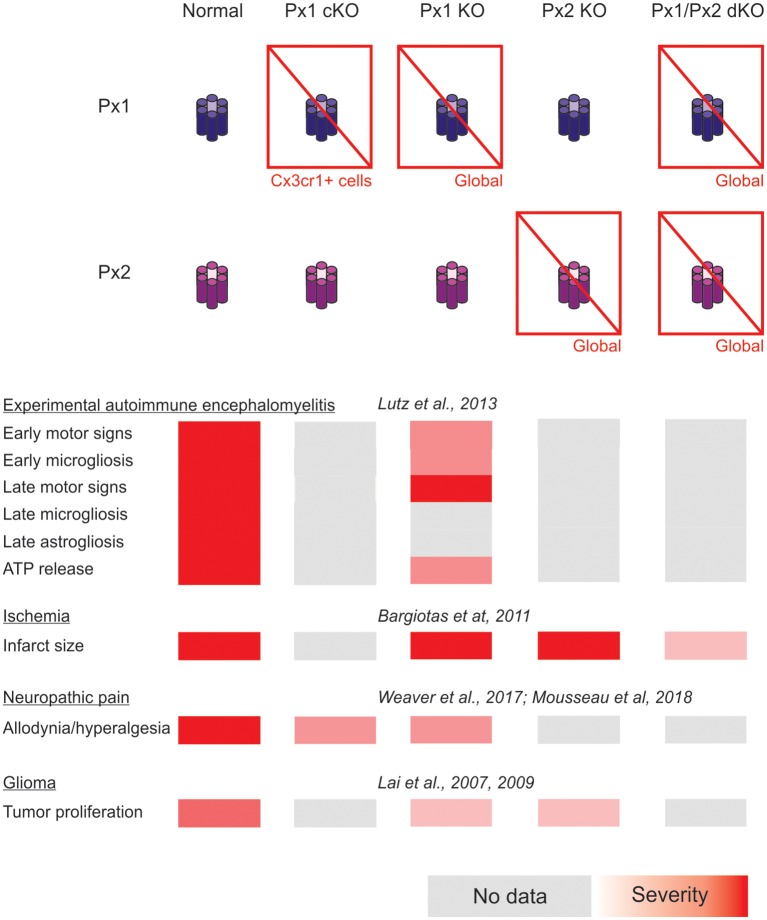
Summary of the impact of global and myeloid cell-selective pannexin function disruption in the context of *in vivo* models of central nervous system disease.

A summary of the cases of connexin- and pannexin-deficient mouse models reviewed is shown in Figures 1, 2. One interesting feature to highlight is that astrogliosis appears dissociated from the availability of functional astrocytic connexins and is likely more related with the inflammatory environment associated with each particular case. The data reviewed gives support to the idea that the hyperactivation of glial hemichannels leads to circuit dysfunction and worsens neurodegeneration in a variety of contexts. The generally beneficial effect of pannexin deficiency vs. the more variable outcomes observed for connexin deficiency supports the idea that connexin proteins can form both hemichannels and gap junctions, while pannexin proteins are predominantly identified with hemichannel function. Efforts to develop blood-brain barrier-permeant connexin hemichannel blockers for systemic administration appear as promising therapeutic tools. Tools to favor the bias of connexins towards gap junction assembly at the expense of hemichannel availability are expected to be beneficial.

Future studies exploiting the advantages of cell-type specific connexin and/or pannexin disruption, time control provided by inducible genetic drivers or vector administration (Table 2) and dissociated manipulation of gap junction channel, hemichannel and non-channel functions will contribute to address the roles of connexins in different nervous system pathologies to better design adequate therapeutic interventions. The focus on cell types whose roles are only beginning to be explored as participants in neuroinflammation (Table 1) is a fertile field for new research. One interesting case is NG2 glia, known to be precursors of mature oligondendrocytes present in numbers comparable to astrocytes in many brain areas (Nishiyama et al., 2005) and to be components of the glial scar after traumatic injury (Schäfer and Tegeder, 2018). Of particular importance is the availability of genetic tools to manipulate NG2 glia selectively (Zhu et al., 2011).

**Table 2:** Genetic tools of potential application to achieve inducible connexin and pannexin manipulations of glial cells *in vivo*.

**Cre-expressing lines**				
**Cell-type selectivity (in nervous system)**	**Construct**	**Induction method**	**RRID**	**Reference**
Astrocytes	Aldh1l1-Cre/ERT2 BAC transgene	Tamoxifen	IMSR_JAX:029655	Srinivasan et al. (2016)
Astrocytes, adult neural progenitors cells	Nestin-CreER transgene	Tamoxifen	IMSR_JAX:012906	Burns et al. (2007)
Astrocytes, adult neural progenitors cells	PAC Fgfr3-icreERT2 transgene	Tamoxifen	IMSR_JAX:025809	Rivers et al. (2008)
Oligodendrocytes, Schwann cells, olfactory ensheathing glia, adult neural progenitors	PLP1-Cre/ERT transgene	Tamoxifen	IMSR_JAX:005975	Doerflinger et al. (2003); Guo et al. (2010) and Piantanida et al. (2019)
NG2 cells (oligodendrocyte and astrocyte precursors)	NG2-Cre/ER™ BAC transgene	Tamoxifen	IMSR_JAX:008538	Zhu et al. (2011)
Microglia, mononuclear phagocyte system	Cx3cr1-Cre/ER transgene	Tamoxifen	IMSR_JAX:020940	Yona et al. (2013) and Zhao et al. (2019)
Microglia, mononuclear phagocyte system	Cx3cr1-Cre/ER/YFP transgene	Tamoxifen	IMSR_JAX:021160	Parkhurst et al. (2013)
Microglia, mononuclear phagocyte system	Iba1-tTA transgene	Doxicycline	IMSR_RBRC05769	Tanaka et al. (2012)
**Cre-responding lines**				
**Cell-type selectivity (in nervous system)**	**Construct**	**Expressed protein/s**	**RRID**	**Reference**
Astrocytes	Aldh1l1-loxP-EGFP-4XpolyA-loxP-diptheria toxin A (DTA)-polyA BAC transgene	EGFP before recombination, DTA after recombination	IMSR_JAX:026033	Tsai et al. (2012)
**Viral vectors**				
**∣rule Cell-type selectivity (in nervous system)**	**Construct**	**Expressed protein/s**	**RRID**	**Reference**
Astrocytes (adult non-neurogenic regions)	AAV(5)-GFAP(2.2)-iCre	Cre recombinase		García-Cáceres et al. (2016)
Astrocytes	AAV-DJ-hALDH1L1-Cre	Cre recombinase		Koh et al. (2017)
**Connexin and pannexin floxed lines**				
**Targeted gene**	**Targeted sequence**	**Targeted connexins**	**RRID**	**Reference**
*Gja1*	Exon 2	Cx43	IMSR_JAX:008039	Liao et al. (2001) and Clasadonte et al. (2017)
*Gjb2*	Exon 2	Cx26		Cohen-Salmon et al. (2002)
*Panx1*	Exon 3/4	Px1		Dvoriantchikova et al. (2012) and Poon et al. (2014)

In addition, forthcoming studies will need to address complex interactions arising from combined selective connexin deficiency in more than one cell type. We should not be surprised by finding regional, age and pathology-dependent differences instead of a more general rule. Figures 1, 2 highlight the complexity of drawing unequivocal conclusions regarding the relevance of hemichannel, gap junction and non-channel functions of connexins and pannexins. Despite the availability of a substantial diversity of genetic tools to distinguish among these functions, it is uncommon to find comparisons of their effects in the same disease model with experiments run in parallel to minimize factors of variability. We envision that collaborative work of different groups with the aim of shedding light on this question will generate invaluable new and confirmative data crucial for the design of new therapies.

## Author Contributions

LB and LR wrote the manuscript. LA and AP revised the manuscript and made important editions.

## Conflict of Interest

The authors declare that the research was conducted in the absence of any commercial or financial relationships that could be construed as a potential conflict of interest.
